# The relationship between serum oestrogen levels and clinical outcomes of hormone replacement therapy-frozen embryo transfer: a retrospective clinical study

**DOI:** 10.1186/s12884-022-04605-2

**Published:** 2022-03-29

**Authors:** Na Kong, Jingyu Liu, Chunxue Zhang, Yue Jiang, Yingchun Zhu, Guijun Yan, Haixiang Sun, Chenyang Huang

**Affiliations:** 1 Reproductive Medicine Center, Nanjing Drum Tower Hospital, The Affiliated Hospital of Nanjing University Medical School, 210008 Nanjing, People’s Republic of China; 2 Center for Molecular Reproductive Medicine, Nanjing University, 210008 Nanjing, China; 3 Drum Tower Clinic Medical College of Nanjing Medical University, 210008 Nanjing, China

**Keywords:** Clinical pregnancy rate, Endometrial transformation, Endometrial thicknesses, Hormone replacement therapy-frozen embryo transfer, Serum oestrogen levels

## Abstract

**Background:**

This study aimed to explore the relationship between serum oestrogen (E_2_) levels before endometrial transformation and pregnancy outcomes of hormone replacement therapy-frozen embryo transfer (HRT-FET) cycles, which has been investigated for years without any consensus.

**Methods:**

A retrospective cohort study of 10,209 cycles HRT-FET cycles was conducted at the Reproductive Medicine Center of Nanjing Drum Tower Hospital from March 2017 to December 2020. A smooth fitting curve was constructed to identify the relationship between serum E_2_ levels before endometrial transformation and the clinical pregnancy rate. Then, threshold and saturation effect analysis was employed to explore the cut-off value of serum E_2_ levels. In addition, patients were divided into 2 groups based on their levels of serum E_2_ measured before progesterone-induced endometrial transformation: Group 1, < 300 pg/mL (*n* = 6251) and Group 2, ≥ 300 pg/mL (*n* = 3958). The clinical pregnancy and miscarriage rates of all groups were compared. Further smooth fitting curve analysis was employed by different subgroups segmented according to different endometrial thicknesses.

**Results:**

When the serum E_2_ level was greater than 300 pg/mL, the clinical pregnancy rate decreased significantly (62.9% vs. 59.8%, *p* < 0.01), but the miscarriage rates were similar (13.5% vs. 15.6%, *p* = 0.14). While serum E_2_ level reached or exceeded 1400 pg/mL, there was no significant correlation between the clinical pregnancy rate and E_2_ level. The clinical pregnancy rate reached its higher level at lower E_2_ levels, regardless of the different endometrail thicknesses.

**Conclusions:**

Patients with a lower pretransformation serum E_2_ level (less than 300 pg/mL) have a higher clinical pregnancy rate and there was no correlation between the clinical pregnancy rate and a higher serum E_2_ level (greater than 1400 pg/mL) in HRT-FET cycles.

**Supplementary information:**

The online version contains supplementary material available at 10.1186/s12884-022-04605-2.

## Background

The use of frozen embryo transfer (FET) cycles has been increasing in recent years, because of the safety and comfort of patients, the decreased risk of ovarian hyperstimulation syndrome (OHSS) and the similar cumulative pregnancy rates compared with fresh embryo transfer cycles [1–5]. The artificial hormone replacement therapy (HRT) cycle for FET is widely used for its stable clinical pregnancy rate and more convenient time schedule [6, 7]. However, several studies suggested that HRT-FET could increase the risk of pregnancy-related complications (hypertension disorders, placenta accrete and intrahepatic cholestasis of pregnancy), as well as the risk of low birth weight and small for gestational age, which was speculated to be related to the abnormal oestrogen (E_2_) level of HRT-FET cycles [8, 9]. Therefore, the control of serum E_2_ level in HRT-FET cycles needs further discussing.

Serum E_2_ is essential for endometrial receptivity, myometrial spiral artery remodelling, and placental development [10]. Studies have shown that higher serum E_2_ levels lead to impaired endometrial receptivity and reduced clinical pregnancy rates [11]. In addition, high serum E_2_ levels before embryo transfer in fresh in vitro fertilization (IVF) cycles are strongly associated with decreased embryo implantation rates [12]. Furthermore, high serum E_2_ levels in IVF cycles increase the incidences of preeclampsia, foetal growth restriction and low-birth-weight infants [13, 14]. Therefore, serum E_2_ levels during the HRT cycle may be closely related to pregnancy outcomes.

Hormone replacement cycles are commonly employed in our reproductive medicine centre, and some patients have serum E_2_ levels that are obviously higher than the natural physiologic levels before endometrial transformation. However, the number of relevant studies on the relationship between serum E_2_ levels before endometrial transformation and pregnancy outcomes of FET cycles is limited to date [11, 15, 16], and these studies have failed to reach a consensus. Therefore, we conducted a retrospective review of recent hormonal replacement FET cycles at the reproductive medicine centre of Nanjing Drum Tower Hospital to explore the relationships between serum E_2_ levels before endometrial transformation and clinical pregnancy outcomes.

## Methods

### Patients

From March 2017 to December 2020, all patients who underwent frozen thawed embryo transfer with artificial hormone replacement (Femoston, 2 mg oestradiol; 2 mg oestradiol with 10 mg dydrogesterone, Abbott, USA) cycles at the reproductive medicine centre of Nanjing Drum Tower Hospital were included in this retrospective study. Patients were 20 to 39 years of age when undergoing HRT-FET and had a body mass index (BMI) of 20.5 to 30.4 kg/m^2^. All patient couples provided written informed consent. All patients received a comprehensive prepregnancy physical examination to exclude drugs and pregnancy contraindications before FET cycles. The general health status of the patients involved in this study are normal. The exclusion criteria for this study were as follows: (1) Use of other hormonal replacement drugs; (2) More than three transfer cycles; (3) Use of gonadotrophin-releasing hormone agonist (GnRHa) pretreatment; (4) Combined hydrosalpinx or lesions of the uterine cavity and endometrium; or (5) Endometriosis or adenomyosis.

### Endometrial preparation and thawed embryo transfer

Patients without abnormalities (sex hormone levels, uterus and adnexa) in HRT-FET cycles were started with oral oestradiol (Femoston, Abbott, USA, 2 mg oestradiol t.i.d. × 14 days) on the second day of their menstrual cycle. Serum E_2_ and P levels and endometrial thickness were monitored. Oestradiol tablets were additionally administered vaginally (Femoston, 2 mg oestradiol q.d.) according to cases of a lower endometrial thickness. When the endometrial thickness met a certain standard, oral oestradiol combined with dydrogesterone compound tablets (Femoston, 2 mg oestradiol and 10 mg dydrogesterone t.i.d. × 5 or 6 days) were administered, along with intramuscular injection of progesterone (P) at 60 mg q.d. for 5 or 6 days to induce endometrial transformation. The serum E_2_ and *P* values mentioned in this manuscript were measured 1 or 2 days before initiating endometrial transformation. At the fifth day of endometrial transformation, cleavage-stage embryos were thawed and transferred. Blastocysts were thawed and transferred at the 6th day of endometrial transformation. All medications were maintained at original doses after embryo transfer. Patients usually take Femoston (2 mg oestradiol and 10 mg dydrogesterone, t.i.d.) and progesterone sustained-release vaginal gel (90 mg, q.d.) for luteal support. Serum β-human chorionic gonadotropin (β-hCG) levels were detected 2 weeks after embryo transfer to determine biochemical pregnancy. Patients with elevated serum β-hCG levels were examined by transvaginal ultrasound 4 weeks after embryo transfer to confirm the clinical pregnancy and number of implanted embryos. Luteal support was maintained for 2 months after transfer when the patient became pregnant. The patient was continuously followed up to identify any abnormalities in pregnancy.

### Statistical analysis

To analyse the effect of serum E_2_ levels before endometrial transformation on the clinical outcomes of HRT-FET cycles, we performed a smooth curve fit analysis. Then, threshold and saturation effect analysis were employed to explore the cut-off value of serum E_2_ levels. Two cut-off values of E_2_ levels were identified. When the serum E_2_ level was less than 300 pg/mL, the clinical pregnancy rate was at a high level and was not related to the serum E_2_ level. When the serum E_2_ level is greater than 300 pg/mL, the clinical pregnancy rate continues to decline. Therefore, cycles were divided into the following 2 groups based on their levels of serum E_2_ measured before endometrial transformation: Group 1, < 300 pg/mL and Group 2, ≥ 300 pg/mL. We used the Kolmogorov-Smirnov normality test to detect the normal distribution of the variables. T-test was employed for the normally distributed variables and Mann Whitney-U test was employed for the the non-normally distributed variables. For the statistical analysis for categorical variables, the variables in Table 1 were tested by chi-squared test (meeting the requirements of chi-square test: theoretical frequency (T) > 5 and sample number (n) > 40). The parameters distributed with normally distribution were explained as Mean ± Standard Deviation (SD) and the parameters distributed with non-normally distribution was explained as Median (25th-75th percentiles). In addition, to analyse the effects of endometrial thickness, the data were divided into two subgroups according to the predicted threshold of endometrial thickness: 8.4 mm. A smooth curve fit analysis of serum E_2_ levels and clinical pregnancy was employed by different endometrial thicknesses (< 8.4 mm and ≥ 8.4 mm). All the analyses were performed with the R package (version 3.6.0) and EmpowerStats (X&Y Solution, Inc., Boston, MA). A *p* value < 0.05 was considered statistically significant.

**Table 1:** Comparison of general characteristics and FET outcomes data between different E_2_ level groups

Variable	Group 1	Group 2	*p* value
**Cases (n)**	6251	3958	
**Baseline characteristics**			
**Age, median (25th-75th percentiles), years**	30.0 (28.0–33.0)	30.0 (28.0–34.0)	0.06
**Body mass index, mean ± SD, kg/m** ^**2**^	24.0 ± 3.3	24.4 ± 3.1	0.42
**No. of cycles, mean ± SD**	2.6 ± 0.9	2.6 ± 0.9	0.43
**FET outcomes**			
**E**_**2**_, **median (25th-75th percentiles), pg/mL**	193.00 (149.00-238.52)	510.07 (371.06-1473.14)	< 0.01
**P, median (25th-75th percentiles), ng/mL**	0.17 (0.07–0.40)	0.34 (0.16–0.59)	< 0.01
**Endometrial thickness**, **median (25th-75th percentiles), mm**	9.50 (8.80–10.50)	9.00 (8.50–10.00)	< 0.01
**No. of transfer embryos, mean ± SD**	1.40 ± 0.49	1.42 ± 0.49	0.06
**Transfer blastocyst rate (%)**	62.3 (3896/6251)	61.5 (2436/3958)	0.43
**Clinical pregnancy rate (%)**	62.9 (3930/6251)	59.8 (2365/3958)	< 0.01
**Implantation rate (%)**	54.6 (4775/8751)	51.3 (2881/5620)	< 0.01
**Miscarriage rates (%)**	13.5 (529/3930)	15.6 (369/2365)	0.14
**Live birth rate (%)**	52.8 (3299/6251)	48.8 (1930/3958)	< 0.01

Patients had a better clinical outcome (pregnancy rate and implantation rate) with a lower E_2_ level (< 300 pg/mL)

*E*_2_oestrogen, *No.* number, *P* progesterone, *SD *standard deviation

## Results

### Smooth fitting curve of serum E_2_ levels and clinical pregnancy rates

**Fig. 1:**
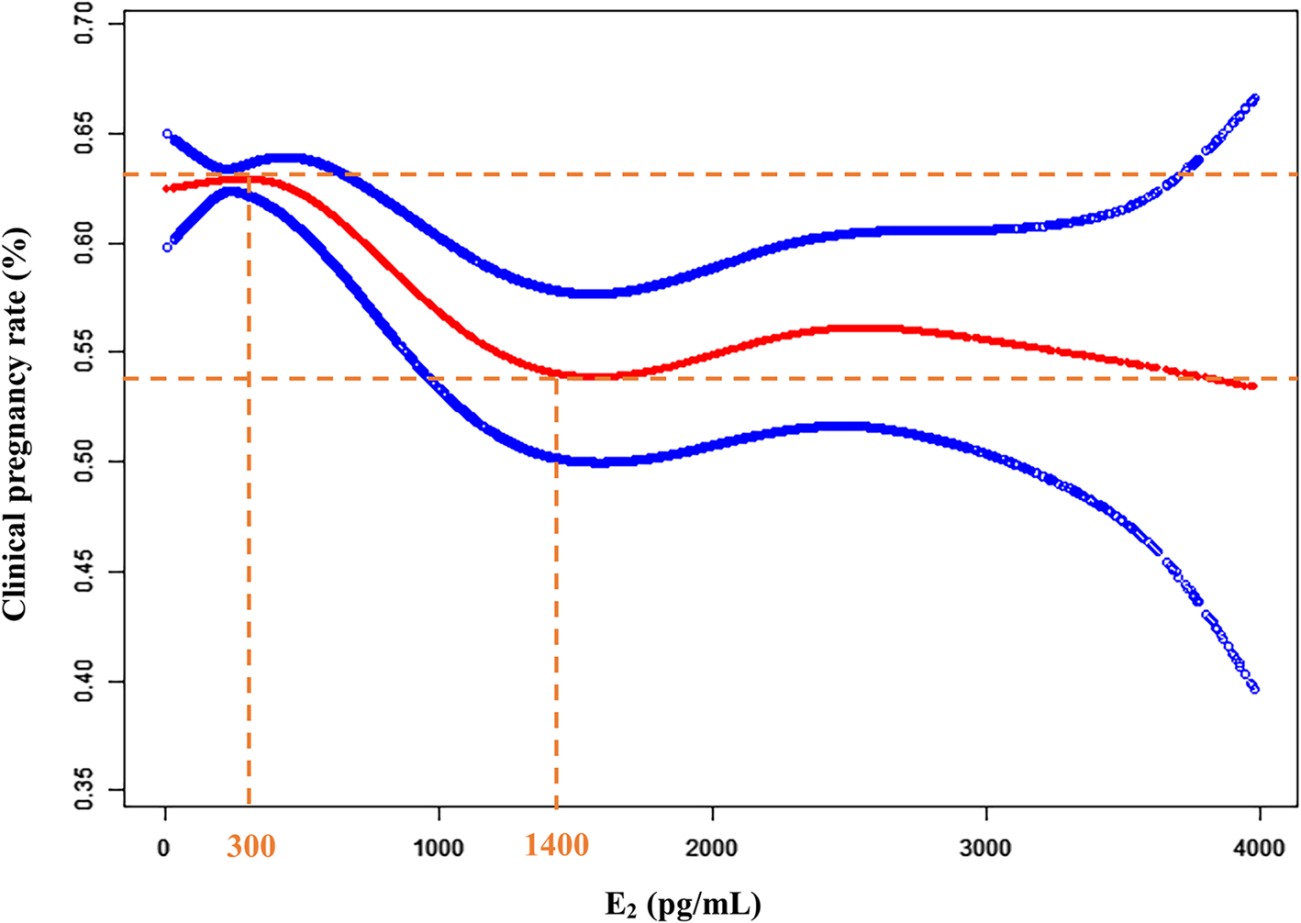
A smooth fitting curve analysis between E_2_ levels and clinical pregnancy rates. The clinical pregnancy rate of the patients decreased obviously as the E_2_ level gradually increased when the E_2_ level was less than 1400 pg/mL

As shown in Fig. 1, the clinical pregnancy rate of the patients decreased obviously as the serum E_2_ level gradually increased. When serum E_2_ levels were less than 300 pg/mL, clinical pregnancy rates were maintained at a higher level. When the serum E_2_ level reached or exceeded 1400 pg/mL, there was no significant change in the clinical pregnancy rate, which was at a lower level (Fig. 1).

### Threshold effect analysis of serum E_2_ levels on the clinical pregnancy rate

In order to clarify the fluctuation of the clinical pregnancy rate with serum E_2_ levels observed in Fig. 1, the results of the threshold effect analysis suggested that there was a curvilinear relationship between serum E_2_ levels and the clinical pregnancy rate (Table 2, logarithmic likelihood ratio = 0.042). When the serum E_2_ level was less than 1400 pg/mL, the clinical pregnancy rate decreased with the increase of the serum E_2_ level (Table 2, OR: 0.975, 95%CI: 0.960–0.989, *p* < 0.001). When the serum E_2_ level reached or exceeded 1400 pg/mL, the clinical pregnancy rate is not affected by the serum E_2_ level (Table 2, OR: 0.999, 95%CI: 0.983–1.015, *p* = 0.907). Therefore, we further performed a threshold effect analysis for the cycles with serum E_2_ levels less than 1400 pg/mL, which suggested that there was no relationship between the clinical pregnancy rate and the serum E_2_ level (Table 3, OR: 1.074, 95%CI: 1.000-1.153, *p* = 0.051) when the serum E_2_ level was less than 300 pg/mL. However, when the serum E_2_ level was between 300 and 1400 pg/mL, the clinical pregnancy rate decreased with the increase of the serum E_2_ level (Table 3, OR: 0.944, 95%CI: 0.919–0.970, *p* < 0.001). A further smooth curve fitting analysis with different serum E_2_ levels also confirmed the results (Fig. S1).

**Table 2:** Threshold effect analysis of E_2_ level (100pg/mL) on the clinical pregnancy rate

Outcome	Clinical pregnancy		
**Model I (linear)**	**OR**	**95% CI**	***p*** **value**
**Linear effect**	0.986	(0.980, 0.992)	< 0.001
**Model II (polyline)**	**OR**	**95% CI**	***p*** **value**
**Predicted threshold (K, E**_**2**_ **level, 100pg/mL)**	14		
**Effect 1 (< K)**	0.975	(0.960, 0.989)	< 0.001
**Effect 2 (> K)**	0.999	(0.983, 1.015)	0.907
**variability of effectiveness**	0.172	(0.019, 0.324)	
**Logarithmic likelihood ratio test**	0.042		

When the E_2_ level was less than 1400 pg/mL, there was a negative relationship between E_2_ levels and the clinical pregnancy rate

*E*_*2* _oestrogen, *OR *odds ratio, *CI *confidence interval, *K *predicted threshold

**Table 3:** Threshold effect analysis of E_2_ level (100pg/mL) in cycles with lower E_2_ level (< 1400 pg/mL)

Outcome	Clinical pregnancy		
**Model I (linear)**	**OR**	**95% CI**	***p*** **value**
**Linear effect**	0.970	(0.950, 0.990)	0.004
**Model II (polyline)**	**OR**	**95% CI**	***p*** **value**
**Predicted threshold (K, E**_**2**_ **level, 100pg/mL)**	3		
**Effect 1 (< K)**	1.074	(1.000, 1.153)	0.051
**Effect 2 (> K)**	0.944	(0.919, 0.970)	< 0.001
**variability of effectiveness**	0.572	(0.499, 0.646)	
**Logarithmic likelihood ratio test**	0.004		

When the E_2_ level was between 300 to 1400 pg/mL, there was a negative relationship between E_2_ levels and the clinical pregnancy rate

*E*_*2* _oestrogen, *OR *odds ratio, *CI *confidence interval, *K *predicted threshold

### General situations of these 2 groups

Furthermore, we divided all enrolled cycles into 2 groups according to different serum E_2_ levels before endometrial transformation: Group 1 - E_2_ < 300 pg/mL and Group 2 - E_2_ ≥ 300 pg/mL. Serum P levels were lower in group 1, and the endometrium was thicker in the first group. There were no significant differences in the number or stages of embryos transferred between the two groups. The clinical pregnancy and embryo implantation rates were much higher in group 1. The early miscarriage rate was similar in these 2 groups. This result suggested that patients might have a better clinical outcome with a lower serum E_2_ level (< 300 pg/mL), but there might be some indefinite factors which might have influence on HRT-FET outcomes, such as patient age, serum P level, and endometrial thickness, among others.

### Analysis of the different endometrial thicknesses affecting the clinical outcomes of HRT-FET

To evaluate whether the endometrial thickness affected the clinical outcomes of HRT-FET, we conducted a threshold effect analysis of the endometrial thickness (Table 4). The results suggested a predicted threshold of endometrial thickness associated with a clinical pregnancy rate of 8.4 mm. In addition, we performed a smooth fitting curve for the relationship between pretransformation serum E_2_ level and clinical pregnancy rate according to the different endometrial thicknesses (< 8.4 mm and ≥ 8.4 mm) of HRT-FET cycles (Fig. S2). Regardless of the levels of endometrial thickness, a decreasing trend in the clinical pregnancy rate was also detected with increasing E_2_ levels before endometrial transformation. This result suggested that the different endometrial thickness levels might not affect the adverse effect of increased serum E_2_ levels on clinical pregnancy of HRT-FET cycles.

**Table 4:** Threshold effect analysis of the endometrial thickness on the clinical pregnancy rate

Outcome	Clinical pregnancy		
**Model I (linear)**	**OR**	**95% CI**	***p*** **value**
**Linear effect**	1.058	(1.029, 1.088)	< 0.01
**Model II (polyline)**	**OR**	**95% CI**	***p*** **value**
**Predicted threshold (K, Endometrial thickness, mm)**	8.4		
**Effect 1 (< K)**	1.757	(1.494, 2.065)	< 0.01
**Effect 2 (> K)**	1.010	(0.979, 1.042)	0.52
**variability of effectiveness**	0.575	(0.483, 0.684)	
**Logarithmic likelihood ratio test**	< 0.01		

The predicted cut-off value of endometrial thickness was 8.4 mm

*OR* odds ratio, *CI *confidence interval, *K *predicted threshold

## Discussion

In our study, the clinical pregnancy rate maintained a high level when the serum E_2_ level before endometrial transformation was less than 300 pg/mL. However, the serum E_2_ level couldn’t affect the clinical pregnancy rate of HRT-FET when the serum E_2_ level is more than 1400 pg/mL.

In 1983, Trounson A first reported a successful pregnancy by FET. FET is currently widely used in clinical human-assisted reproductive technology (ART) [17], mainly for patients who have a previously failed fresh embryo transfer, whose fresh embryo transfer was cancelled due to the risk of OHSS, or who require embryo storage for other reasons [18]. Artificial hormone replacement cycles have been more widely employed because of their convenience. Exogenous oestrogen and progestogen are orally administered to change the endometrium to achieve synchronization with the embryos, which is necessary for embryo implantation in HRT-FET cycles. Embryo implantation is one of the central factors in ART and depends mainly on the ability of the endometrium to receive the embryo for implantation and on the quality of the embryo. Endometrial growth in HRT-FET cycles relies on exogenous oestrogens taken by the patients, and there are two regimens: fixed or escalating doses. Considering patient compliance, the HRT-FET cycles we included in this study were all performed with a fixed oestrogen dose. The serum E_2_ and P levels and endometrial thickness of the patients were monitored regularly, and progesterone was administered to transform the endometrium when the endometrium reached the expected thickness. However, there are differences in the absorption and metabolism of exogenous oestrogens among different individuals. Therefore, there are also differences in serum E_2_ levels among different individuals. Of interest is whether the difference in serum E_2_ levels influenced the clinical outcomes of HRT-FET cycles. Some studies have explored the association between serum E_2_ levels before endometrial transformation and the clinical outcomes of HRT-FET cycles, but they remain inconclusive [11, 15, 16, 19]. In our retrospective study, the results suggested that the levels of serum E_2_ before endometrial transformation were closely related to the clinical pregnancy rates of patients with HRT-FET cycles when they were less than 1400 pg/mL. Higher clinical pregnancy and embryo implantation rates were achieved when serum E_2_ levels were less than 300 pg/mL.

Although sustained elevations in oestrogen in the follicular phase are indispensable for endometrial growth, previous studies have suggested that excessive oestrogen may have adverse effects. In vitro studies reported that oestrogen overexpression in first-trimester human trophoblast cells and the first-trimester placenta is able to inhibit trophoblast invasion by inducing apoptosis, potentially leading to abnormal pregnancy outcomes [20]. In addition, a number of in vivo studies have explored the effects of serum E_2_ levels on endometrial function. In mouse models, oestrogen should be maintained in a certain range to enable the uterus to be receptive, and properly increased serum E_2_ levels are closely associated with altered expression of genes involved in embryo implantation [21]. At the same time, in a baboon model, higher serum E_2_ levels during early pregnancy (first 60 days) allowed extra villous trophoblast invasion and uterine artery-related functions affecting the development of pregnancy [21]. It follows that appropriate serum E_2_ levels have important effects on both embryo implantation and ongoing pregnancy but that serum E_2_ levels that are too high may adversely affect it. Finding a reasonable range of serum E_2_ levels before endometrial transformation in HRT-FET cycles is essential to improve the pregnancy rate of ART cycles [22]. Clinically, excessive serum E_2_ levels after controlled ovarian hyperstimulation (COH) during IVF cycles may lead to a reduced clinical pregnancy rate [23] or adverse pregnancy outcomes [13, 24]. In a previous study, when serum E_2_ levels reached 3560 ± 1233 pg/mL or even higher [25] on the hCG trigger day in fresh transplant cycles, the clinical pregnancy rate was significantly lower. However, serum E_2_ levels in fresh transplant cycles are much higher than those in HRT-FET cycles in most situations. Therefore, this serum E_2_ limit is of limited significance for artificial hormone replacement cycle guidance. There are many studies focusing on HRT-FET cycles that have set cut-off values for serum E_2_ levels before endometrial transformation: 299, 400, 600 or 689 pg/mL [11, 16, 26]. Our study was not a direct equivalent of previous studies. In our retrospective study, a smooth curve fitting model was innovatively used to analyse HRT-FET data from our reproductive medicine centre over a period of nearly 3 years, suggesting that the peak clinical pregnancy rate occurs when the serum E_2_ level before endometrial transformation is less than 300 pg/mL. Regarding this, we grouped serum E_2_ [12, 27, 28] levels before endometrial transformation, and further statistical analysis suggested that we could achieve better clinical pregnancy outcomes when serum E_2_ levels were lower than 300 pg/mL in HRT-FET cycles. It is generally accepted that a high serum E_2_ level in an FET cycle refers to more than a peak (284.5 ± 77.9 pg/mL) value in the physiological state of the natural cycle [21]. In addition, the serum E_2_ level of patients in group 1 (E_2_ < 300 pg/mL) of our enrolled cycle was 194.11 ± 56.84 pg/mL, which was closer to the natural cycle situation, which had less of an effect on endometrial receptivity.

According to further observation of the initial fitting curve result, we found that when the serum E_2_ level gradually increased to a certain extent, the clinical pregnancy rate had a slight fluctuation. There was a slightly positive correlation between increasing serum E_2_ levels and the clinical pregnancy rate when the serum E_2_ level was less than 300 pg/mL and within a certain range (approximately 1400–2400 pg/mL). Conversely, when the serum E_2_ level was in a certain range (300–1400 pg/mL) and more than 2400 pg/mL, the expected clinical pregnancy rate decreased progressively with increasing serum E_2_ levels. The serum E_2_ increase in the HRT-FET cycles of this study far exceeded the normal physiological category, which was due mainly to 2 mg of oestradiol for the vaginal plug added in patients with unexpected endometrial thickness. It has been reported that in vaginal plugs with oestradiol 4 mg/day, the serum E_2_ concentration can reach a maximum of 4800 pg/mL; in addition, the combination of oral and vaginal oestradiol can achieve better endometrial thickness and improve endometrial receptivity [29]. Therefore, most of the patients in the HRT-FET cycles with higher serum E_2_ levels (> 1400 pg/mL) had oestradiol medication vaginally because of an unexpected endometrial thickness. Higher serum E_2_ levels may be better for endometrial proliferation, such that clinical pregnancy rates improve with higher serum E_2_ levels when E_2_ levels are between approximately 1400–2400 pg/mL. When serum E_2_ levels reach a certain range, they will have a limited effect on the improvement of endometrial thickness. When serum E_2_ levels are greater than approximately 2400 pg/mL, the clinical pregnancy rate will decrease with higher serum E_2_ levels. These results are valuable for the regulation of oestrogen dosage during HRT-FET cycles in our reproductive medicine centre.

In IVF-ET cycles, endometrial thickness can reflect the functional status of the endometrium to a certain degree [30]. Appropriate endometrial thickness is an essential condition for embryo implantation. Endometrial thickness is a routine detection index to evaluate the ability to accept embryo implantation because of the convenience and maturity of the measuring procedure. At present, most studies believe that endometrial thickness less than 6–8 mm may lead to adverse clinical outcomes [31]. In our study, there was a significant difference in endometrial thickness between the two groups. Patients with high serum E_2_ levels were mainly caused by vaginal medication, and the main reason for vaginal medication is the thin endometrial thickness. Therefore, the endometrial thickness of group 2 was slightly lower than that of group 1. To exclude the influence of endometrial thickness, we further used the threshold prediction model to calculate the cut-off value (8.4 mm) that might affect the clinical outcomes. On this basis, we divide the research data into two subgroups for smoothing curve fitting again. The results showed that the clinical pregnancy rate decreased with increasing serum E_2_ levels before endometrial transformation, regardless of endometrial thickness. In addition, we also found the difference of serum P levels between these two groups. There are few studies on the impact of serum P level on the clinical pregnancy outcome of HRT-FET cycles, which mainly discuss the impact of serum P level after endometrial transformation or on the day of embryo transfer. Furthermore, we conducted a univariate analysis of the serum P level before transformation, and the results showed that it has no significant effect on the clinical pregnancy rate (Table. S1).

The results of our study are not identical to those of some previous studies. Niu et al. [16] retrospectively reviewed 274 FET cycles. Patients with different serum E_2_ levels on the start day of progesterone had similar pregnancy rates. However, the higher E_2_ level in their study (299 ± 48.9 pg/mL) was much lower than the higher serum E_2_ level in our study. Moreover, the previous study included only the outcomes of cleavage-stage embryo transfer. Celik et al. conducted a retrospective study [28] of 468 patients in 2019: Serum E_2_ monitoring prior to progesterone administration could not predict patient live birth rates. A novel retrospective study [32] suggested no significant difference in FET clinical outcomes when serum E_2_ levels were between 100 and 500 pg/mL before endometrial transformation but that the spontaneous abortion rate was significantly increased when the serum E_2_ level was below 100 pg/mL or over 500 pg/mL. However, the highest serum E_2_ cut-off value of this study was only 500 pg/mL, which was much lower than the high serum E_2_ cut-off value of our study, and this study included only the outcomes of blastocyst transfer. The retrospective analysis in our centre has a larger sample size than previous studies and incorporates different numbers and types of embryos transferred. To control for the influence of embryonic factors on the clinical outcomes of HRT-FET cycles, we further stratified the statistics for the different types of embryos transferred. A smooth curve fit was employed between the level of serum E_2_ before endometrial transformation and the clinical pregnancy rate of patients with different numbers and types of embryos transferred, and we found that the clinical pregnancy rates of the different numbers and types of embryos transferred decreased gradually as the level of serum E_2_ increased (Fig. S3). The results suggested that the increased serum E_2_ level before endometrial transformation impaired the clinical pregnancy rate regardless of the number and types of embryos transferred.

Our study was limited to HRT-FET cycles without pretreatment. However, a larger number of patients were pretreated with GnRHa before oral exogenous oestrogen and progesterone at our centre, and the relationship between pretransformation serum E_2_ levels and clinical outcomes in such patients needs further exploration. In addition, we didn’t include the duration of oestrogen used of patients in HRT-FET cycles, which might has influence on the clinical pregnancy outcomes. The main drawback of this study is its retrospective design. To further clarify the effect of serum E_2_ levels before endometrial transformation on clinical outcomes in HRT-FET cycles, higher-quality and large-scale randomized controlled trials are needed. We can further design clinical randomized controlled studies to clarify the impact of serum E_2_ level on clinical outcome under different method and dose of exogenous oestrogen administration. Therefore, the dosage of exogenous oestrogen can be reduced to avoid drug abuse and drug-related risks while maintaining a high clinical pregnancy rate.

## Conclusions

In summary, when the serum E_2_ level before endometrial transformation was less than 1400 pg/mL, the serum E_2_ level affects the clinical pregnancy rate in the HRT-FET cycle. When the pretransformation serum E_2_ level is less than 300 pg/mL, patients with HRT-FET cycles may achieve a higher possibility of clinical pregnancy.

## Supplementary Information

**Additional file 1.**

**Additional file 2.**

## Data Availability

The datasets generated and analysed during the current study are not publicly available due to the special requirements of our hospital and our reproductive medicine center for the disclosure of patients’ clinical data but are available from the corresponding author on reasonable request.

## Abbreviations

E_2_: oestrogen
HRT-FET: hormone replacement therapy-frozen embryo transfer
P: progesterone
IVF: in vitro fertilization
BMI: body mass index
GnRHa: gonadotrophin-releasing hormone agonist
β-hCG: β-human chorionic gonadotropin
ART: assisted reproductive technology
COH: controlled ovarian hyperstimulation
SD: Standard Deviation
