# Supplementary material for: The relationship between serum oestrogen levels and clinical outcomes of hormone replacement therapy-frozen embryo transfer: a retrospective clinical study
Source: BMC Pregnancy Childbirth. 2022 Mar 29;22:265. doi: 10.1186/s12884-022-04605-2 (PMC8966331; doi:10.1186/s12884-022-04605-2)
Supplement: Supplementary file 1 — Additional file 1. [file 12884_2022_4605_MOESM1_ESM.docx]

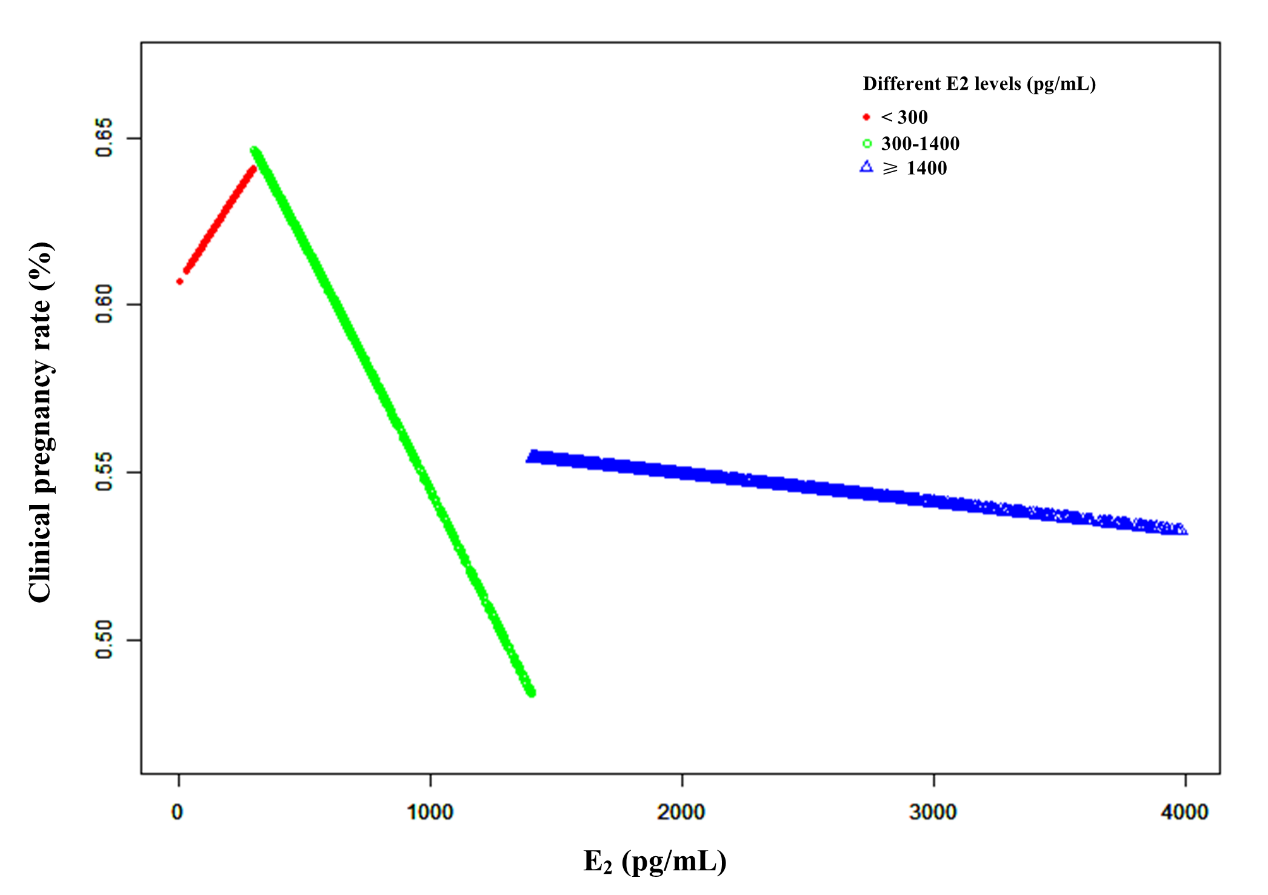


**Figure S1. A smooth fitting curve analysis between E_2_ levels and clinical pregnancy rates with different E_2_ levels.**

The clinical pregnancy rate reached a higher level when the E_2_ level was less than 300 pg/mL, and it decreased with the increase of E_2_ level when the E_2_ level was between 300-1400 pg/mL.


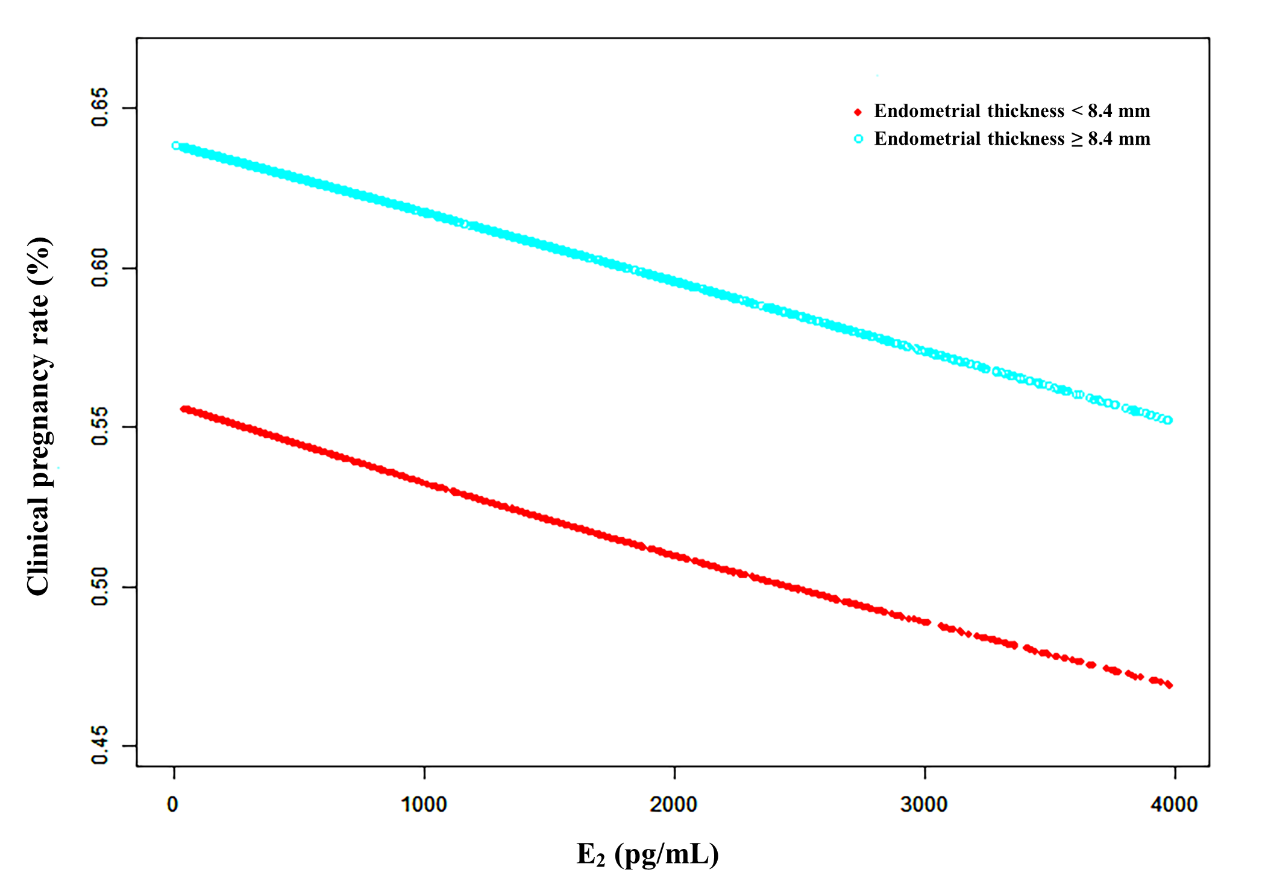


**Figure S2. A smooth fitting curve analysis between E_2_ levels and clinical pregnancy rates based on the different endometrial thicknesses.**

The clinical pregnancy rate of the patients decreased obviously as the E_2_ level gradually increased regardless of the endometrial thickness.


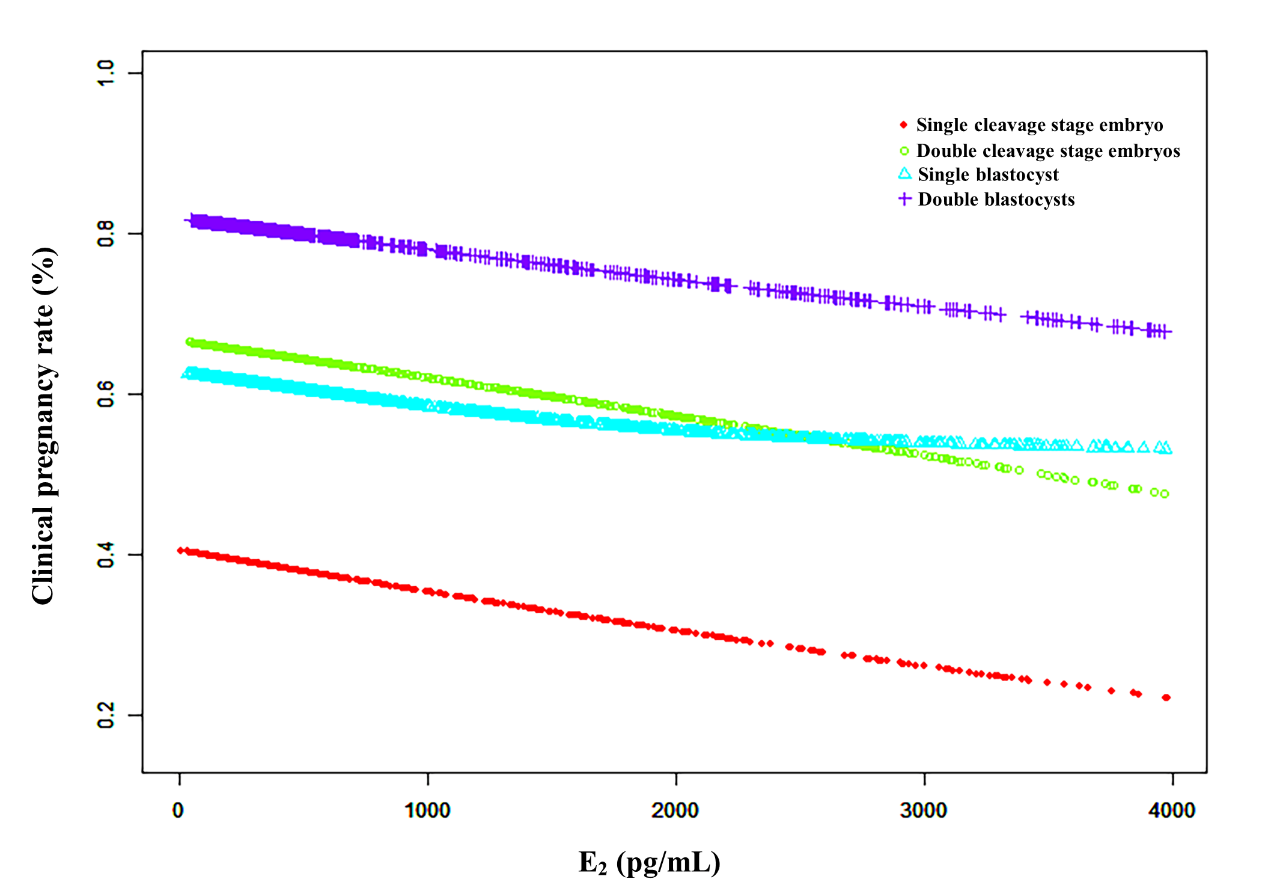


**Figure S3. A smooth fitting curve analysis between E_2_ levels and clinical pregnancy rates.**

The clinical pregnancy rate of the patients decreased obviously as the E_2_ level gradually increased regardless of the numbers and types of embryos transferred.
