# Supplementary material for: The relationship between serum oestrogen levels and clinical outcomes of hormone replacement therapy-frozen embryo transfer: a retrospective clinical study
Source: BMC Pregnancy Childbirth. 2022 Mar 29;22:265. doi: 10.1186/s12884-022-04605-2 (PMC8966331; doi:10.1186/s12884-022-04605-2)
Supplement: Supplementary file 2 — Additional file 2. [file 12884_2022_4605_MOESM2_ESM.docx]

**Table S1. Univariate analysis of the** **serum P level before transformation on the clinical pregnancy rate.**

|  | **Group 1** | | | **Group 2** | | | **Total** | | |
| --- | --- | --- | --- | --- | --- | --- | --- | --- | --- |
|  | **OR** | **95% CI** | **p value** | **OR** | **95% CI** | **p value** | **OR** | **95% CI** | **p value** |
| **P level** | 0.98 | (0.89, 1.08) | 0.66 | 0.83 | (0.71, 1.02) | 0.06 | 0.93 | (0.86, 1.03) | 0.08 |

P, progesterone; OR, odds ratio; CI, confidence interval.

The serum P level before endometrial transformation has no significant effect on the clinical pregnancy rate.
